# Development of a prognostic model for hepatocellular carcinoma using genes involved in aerobic respiration

**DOI:** 10.18632/aging.203021

**Published:** 2021-04-26

**Authors:** Jiawei Rao, Xukun Wu, Xiaozhuan Zhou, Ronghai Deng, Yi Ma

**Affiliations:** 1 Organ Transplant Center, The First Affiliated Hospital, Sun Yat-Sen University, Guangzhou 510080, China; 2 Guangdong Provincial Key Laboratory of Organ Donation and Transplant Immunology, The First Affiliated Hospital, Sun Yat-Sen University, Guangzhou 510080, China; 3 Guangdong Provincial International Cooperation Base of Science and Technology (Organ Transplantation), The First Affiliated Hospital, Sun Yat-Sen University, Guangzhou 510080, China; 4 Department of Hepatology Surgery, The First Affiliated Hospital of Sun Yat-Sen University, Guangzhou, China; 5 Department of Gastroenterology, The First Affiliated Hospital of Sun Yat-Sen University, Guangzhou, China

**Keywords:** hepatocellular carcinoma, prognostic model, oxidative phosphorylation, tricarboxylic acid cycle

## Abstract

Hepatocellular carcinoma (HCC) is the second leading cause of cancer-related death worldwide. Currently, recent risk stratification has only focused on liver function and tumor characteristics. Thus, the purpose of this study was to develop a prognostic model based on genes involved in aerobic respiration. Matched tumor and normal tissues from TCGA and ICGC cohorts were analyzed to identify 15 overlapping differential expressed genes. Cox univariate analysis of the 15 genes in the TCGA cohort revealed they were all associated with disease-specific survival (DSS) in HCC patients. Using LASSO estimation and the optimal value for penalization coefficient lambda 12 genes were selected for the prognostic model, and then HCC patients in the TCGA cohort were dichotomized into low-risk and high-risk groups. Univariate and multivariate Cox analysis demonstrated patients in low-risk group had better survival. Validation of the risk score model with the ICGC cohort produces results consistent with those of the TCGA cohort. In conclusion, this study developed and validated a prognostic model of HCC through a comprehensive analysis of genes involved in aerobic respiration. This model may help develop personalized treatments for patients with HCC.

## INTRODUCTION

Hepatocellular carcinoma (HCC) is the second leading cause of cancer-related death worldwide, and accounts for around 800,000 deaths yearly [3]. HCC is a heterogeneous disease with a number of risk factors including hepatitis B virus (HBV) infection, hepatitis C virus (HCV) infection, obesity, and alcohol abuse. Treatments for HCC include hepatectomy, liver transplantation, radiotherapy, transcatheter arterial chemoembolization (TACE), and chemotherapy [4]; however, the survival of patients with advanced HCC is poor. The assessment of prognosis is important for patients with HCC, as an adequate assessment can prevent low-risk patients from receiving unnecessary treatments, and high-risk patients from relapse or metastasis due to inadequate treatment. Current well-known staging strategies such as tumor, node, metastasis (TNM) and the Barcelona Clinic Liver Cancer (BCLC) system only focus on tumor diameter, tumor metastasis, and liver function. However, the important role of metabolic subtypes is ignored. Thus, the addition of genetic factors to traditional prognostic models may improve risk assessment.

Oxidative phosphorylation (OXPHOS) and the tricarboxylic acid cycle (TCA) in mitochondria are important methods for the production of ATP, and OXPHOS accounts for 80% of the ATP production in mammalian cells. In the 1920s, Otto Warburg observed that cancer cells have high glucose consumption and high lactate production, even in the presence of abundant oxygen, indicating a switch from OXPHOS to glycolysis [1]. The switch may be due to the faster rate of ATP production by glycolysis, which supports the rapid proliferation of malignant cells [2]. To facilitate glycolytic efficiency, the activity of pyruvate dehydrogenase (PDH) is usually suppressed which prevents pyruvate from being transformed to acetyl coenzyme A (acetyl-CoA) for subsequent aerobic mitochondrial metabolism [5]. Thus, the TCA cycle in cancer cells is bypassed. Defects of OXPHOS might be associated with decrease levels of complex I or autophagic degradation, which is caused by oncogenic K-Ras activation [6, 7]. Lee et al. reported that exogenous lactate released by cancer cells can inhibit OXPHOS in normal cells by reducing MRPL13 expression, thus forming a vicious circle [5].

A number of studies have confirmed the relations between OXPHOS, the TCA cycle, and HCC. A study showed that inactivation of OXPHOS, rather than increased activation of glycolysis, was responsible for both inherent and acquired sorafenib resistance of HCC cells [8]. Another study showed that citramalic acid in tumor tissues of HCC patients who were HBV surface (HBsAg) positive was significantly lower than in patients who were HBsAg negative, which indicated an association of TCA cycle suppression and inflammatory status [9].

The TCA cycle and OXPHOS are tightly controlled by the expression levels of associated genes; however, studies have not examined the relations of expression profiles of these genes and HCC prognosis. Thus, the objective of this study was to develop a HCC prognostic model based on the expression of genes associated with the TCA cycle and OXPHOS.

## RESULTS

### Identification of DEGs associated with aerobic respiration

**Figure 1:**
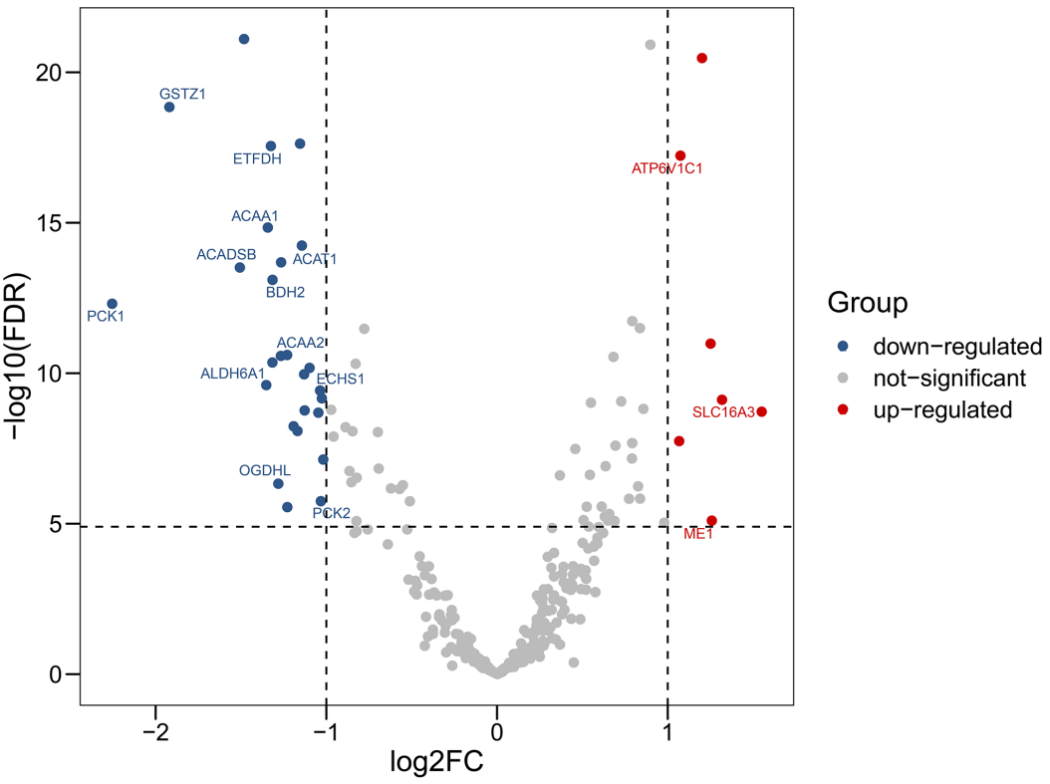
**Volcano plot showing differentially expressed genes in aerobic respiration between hepatocellular carcinoma and normal tissues.** Red dots represent significantly up-regulated genes, blue dots represent significantly down-regulated genes, and gray dots represent no differences between genes.

**Figure 2:**
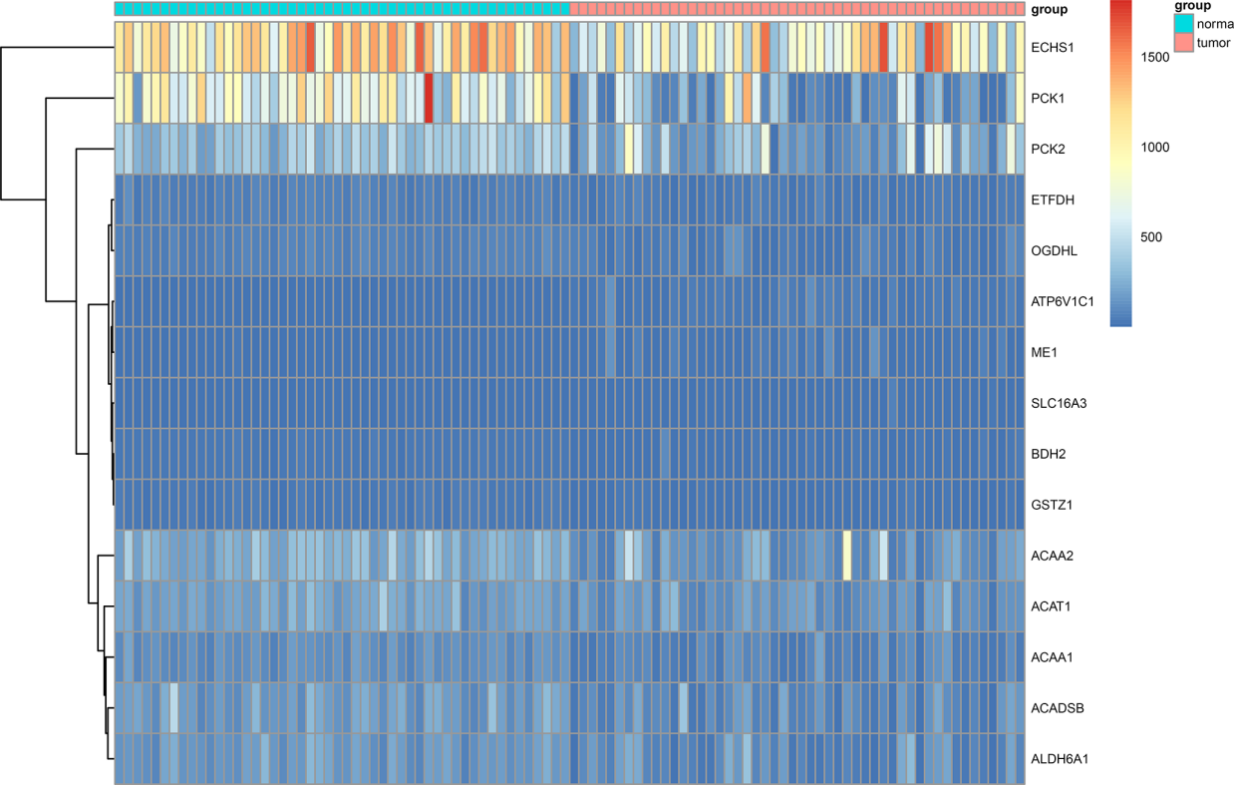
Heat map showing differentially expressed genes in aerobic respiration between 50 matched hepatocellular carcinoma and normal tissue pairs in the TCGA cohort.

We obtained RNA-sequencing data of 50 paired HCC tissues and adjacent normal tissues from TCGA. RNA-sequencing data of 203 HCC tissues and 201 matched adjacent normal tissues from ICGC were also included. Using a threshold |log2 fold-change| > 1.0 and false discovery rate (FDR) adjusted to *P* < 0.05, 3 up-regulated genes (*ATP6V1C1*, *SLC16A3*, *ME1*) and 12 down-regulated genes (*GSTZ1*, *ETFDH*, *ACAA1*, *ACADSB*, *ACAT1*, *BDH2*, *PCK1*, *ACAA2*, *ALDH6A1*, *ECHS1*, *OGDHL*, *PCK2*) were identified (Figure 1, Figure 2).

### Functional enrichment analysis of the DEGs

**Figure 3:**
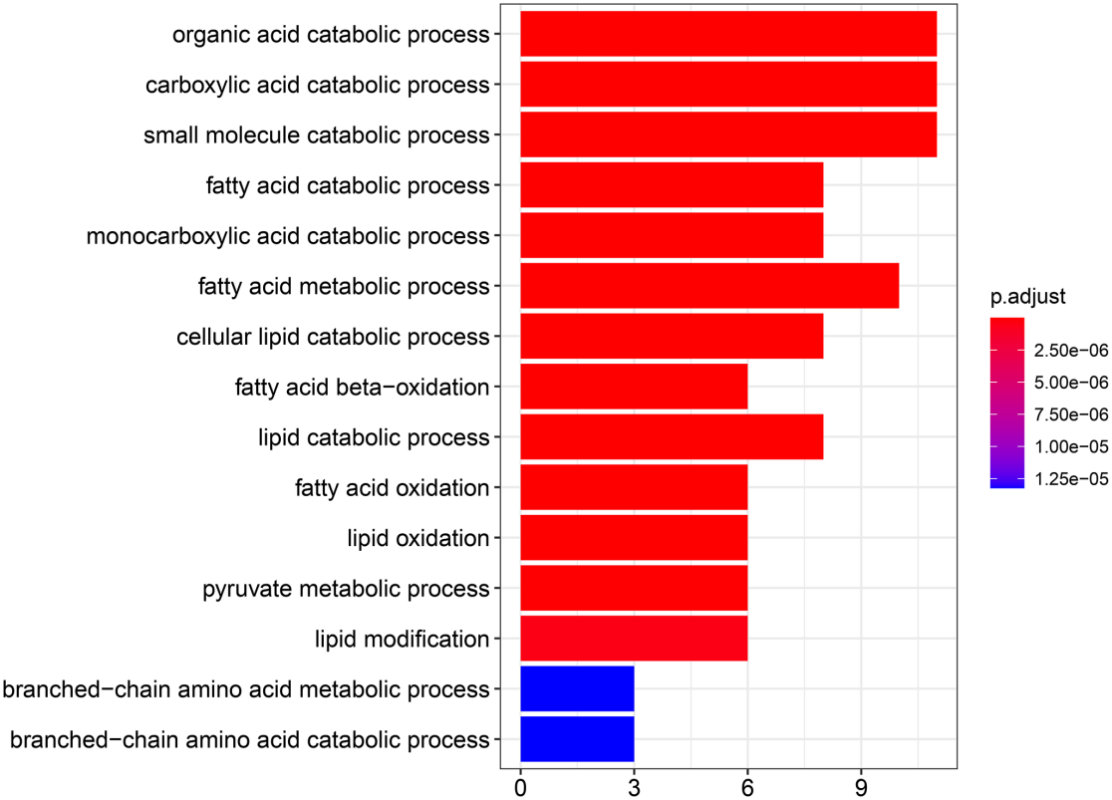
GO analysis showing the biological processes and molecular functions associated with the differentially expressed genes in aerobic respiration.

**Figure 4:**
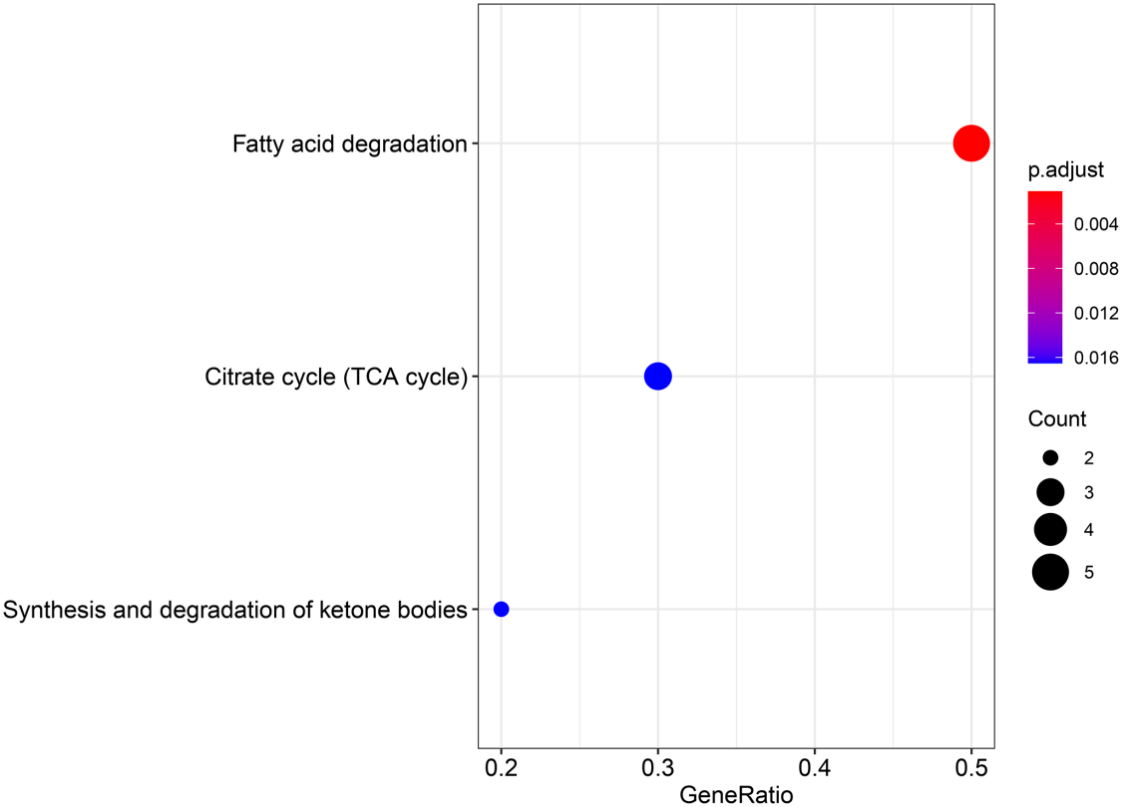
KEGG analysis showing the signaling pathway involved in the differentially expressed genes in aerobic respiration.

**Figure 5:**
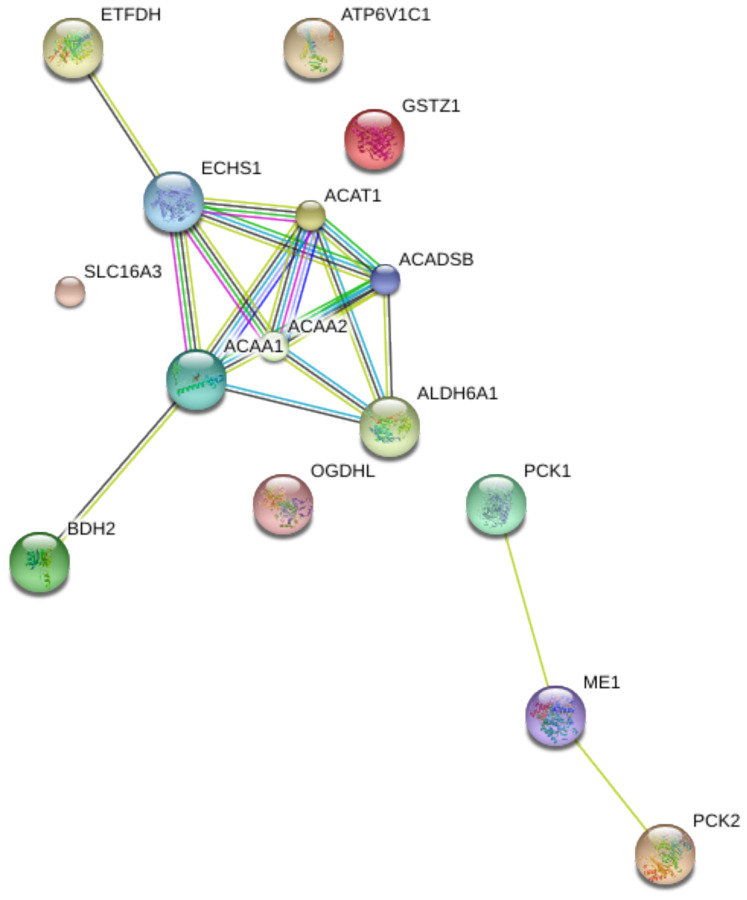
Protein-protein interaction (PPI) network of 15 differentially expressed genes in aerobic respiration.

To explore the biological function of the 15 genes, we conducted functional enrichment analysis via GO annotation and KEGG pathway analyses. Top 30 genes identified by GO analysis are shown in Figure 3, and the results demonstrated that the most frequent biological process category was organic acid catabolic processes. The top 30 enrichment pathways are summarized in Figure 4. The results indicated that the most enriched pathway was fatty acid degradation. Protein-protein network analysis showed that *ACAA1*, *ACAA2*, *ECHS1*, *ACAT1* and *ALDH6A1* were the core genes (Figure 5).

### Development of a prognostic model and model validation

**Figure 6:**
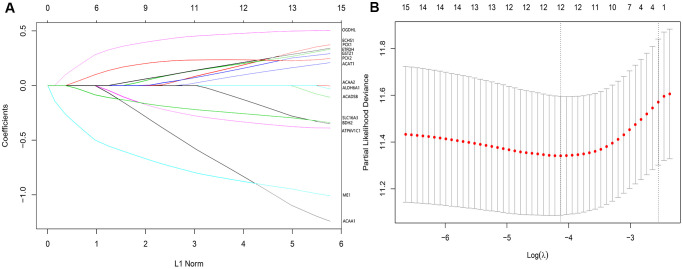
(**A**) LASSO coefficient profiles of the 12 genes of high prognostic value. (**B**) The optimal values of the penalty parameter were determined by 10-fold cross-validation.

In the 357 patients from TCGA cohort, univariate Cox regression analysis indicated that 15 genes were significantly related to DSS. LASSO Cox regression analysis indicated that 12 of the genes had maximum prognostic value (Figure 6A, 6B). These genes were then used to develop a risk score model to predict the prognostic of HCC patients.

Riskscore = *ACAA1* × (–0.8576835) + *ACAT1* × 0.1040123 + *ATP6V1C1* × (–0.3383703) + *BDH2* × (–0.1431161) + *ECHS1* × 0.1992799 + *ETFDH* × 0.2083975 + *GSTZ1* × 0.1662454 + *ME1* × (–0.8860555) + *OGDHL* × 0.4802452 + *PCK1* × 0.2184216 + *PCK2* × 0.2346868 + *SLC16A3* × (–0.2621411).

**Figure 7:**
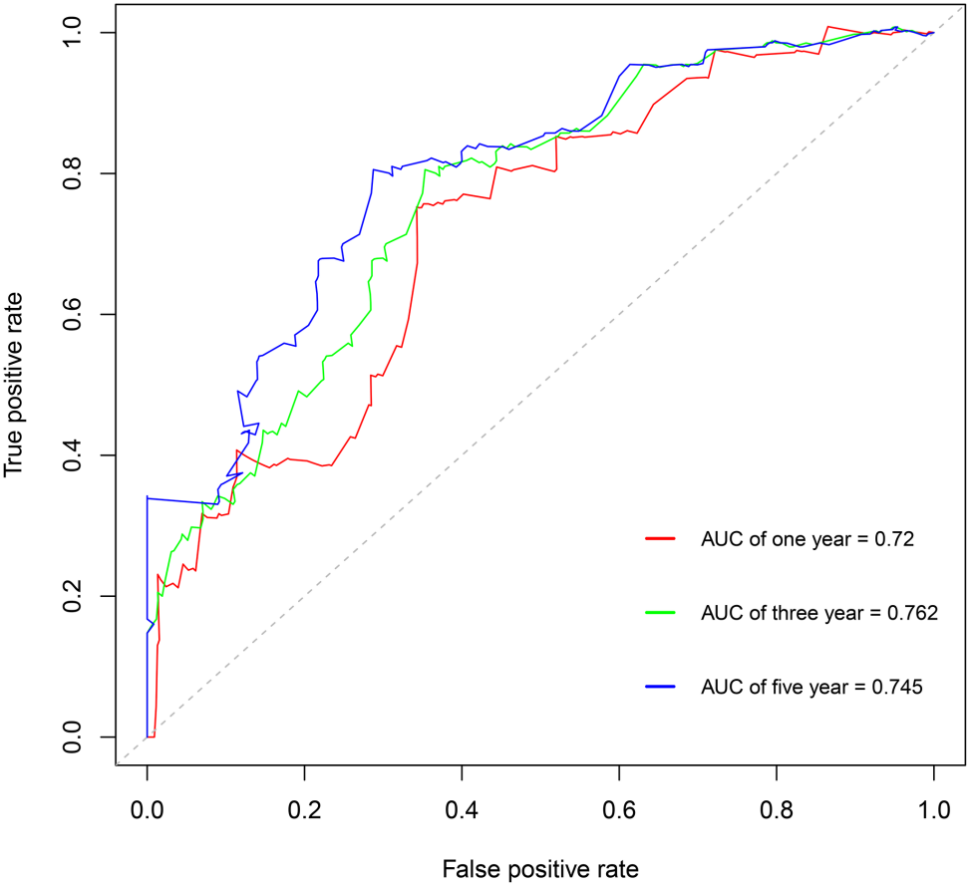
Survival-dependent receiver operating characteristic (ROC) curves showing the prognostic value of the model.

**Figure 8:**
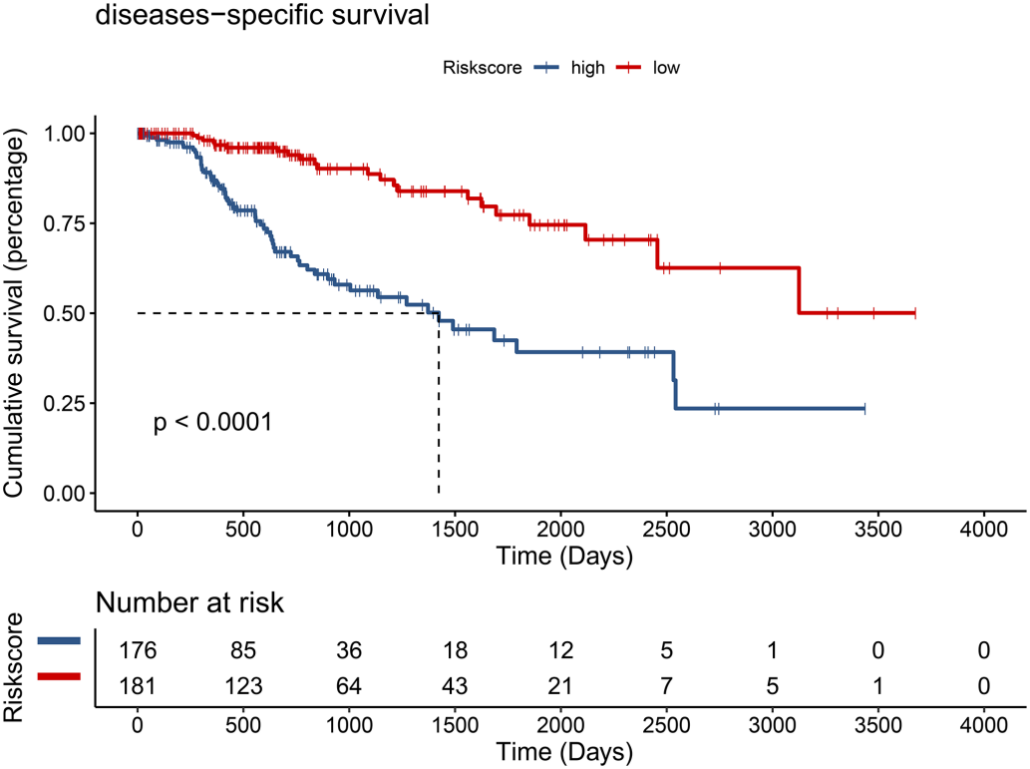
Hepatocellular carcinoma patients from the TCGA cohort in the low-risk group had better disease-specific survival (DSS) (*P* < 0.001).

**Figure 9:**
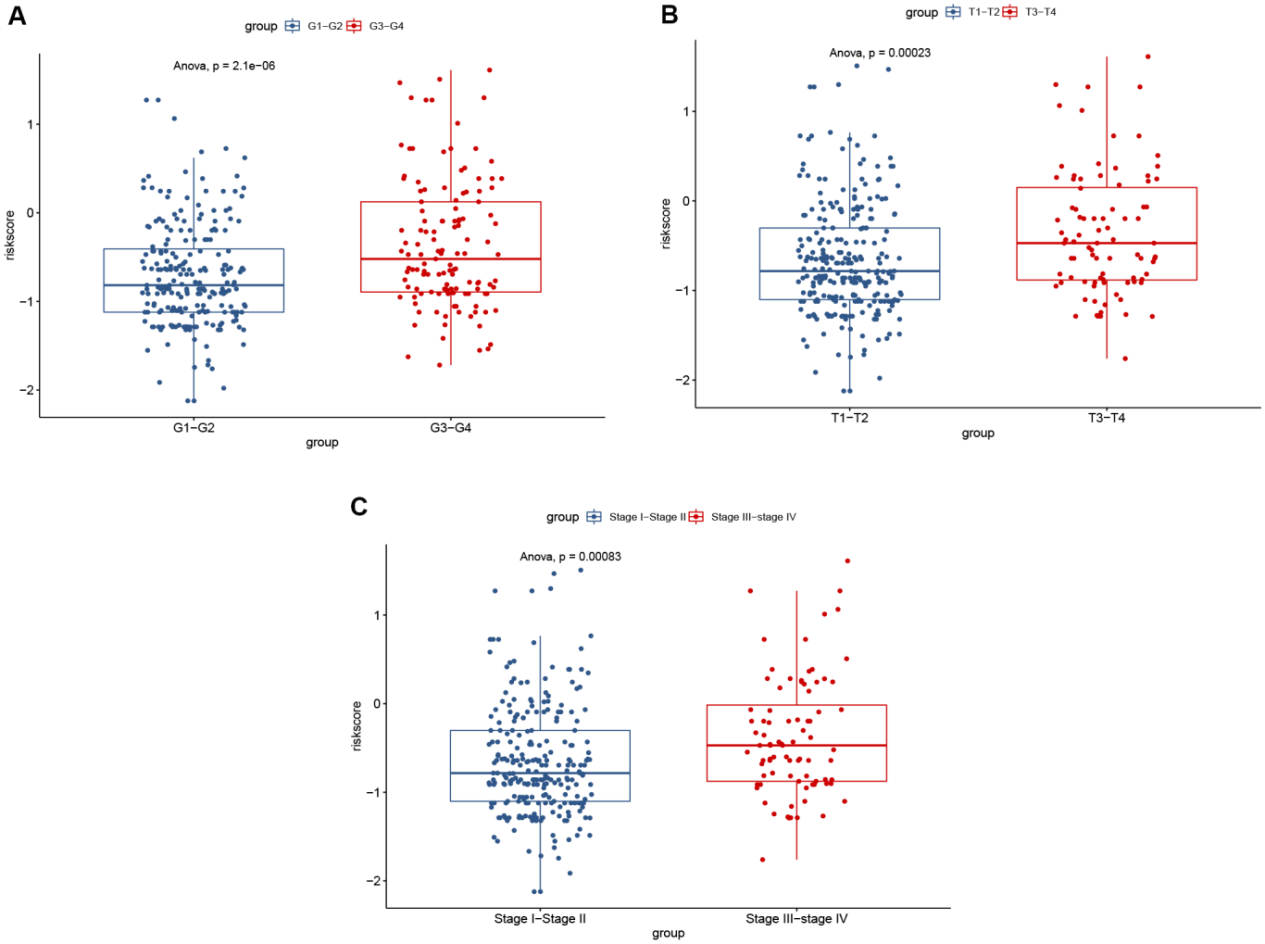
Significant differences of risk score were found between: (**A**) different tumor grades; (**B**) different T stages; and (**C**) different AJCC stages.

Using the risk score, patients from TCGA were categorized as high-risk and low-risk. The area under the receiver operating characteristic (ROC) curve (AUC) of the prognostic model for DSS was 0.72 at 1 year, 0.762 at 3 years, and 0.745 at 5 years (Figure 7). Kaplan-Meier analysis showed a significant survival difference between the 2 groups (*P* < 0.001) (Figure 8). The riskscore was significantly higher (*P* < 0.05) in patients with higher grade tumors (Figure 9A), more advanced T stage (Figure 9B), and more advanced American Joint Committee on Cancer (AJCC) stage (Figure 9C), but was not associated with HBV status (Supplementary Figure 1).

**Figure 10:**
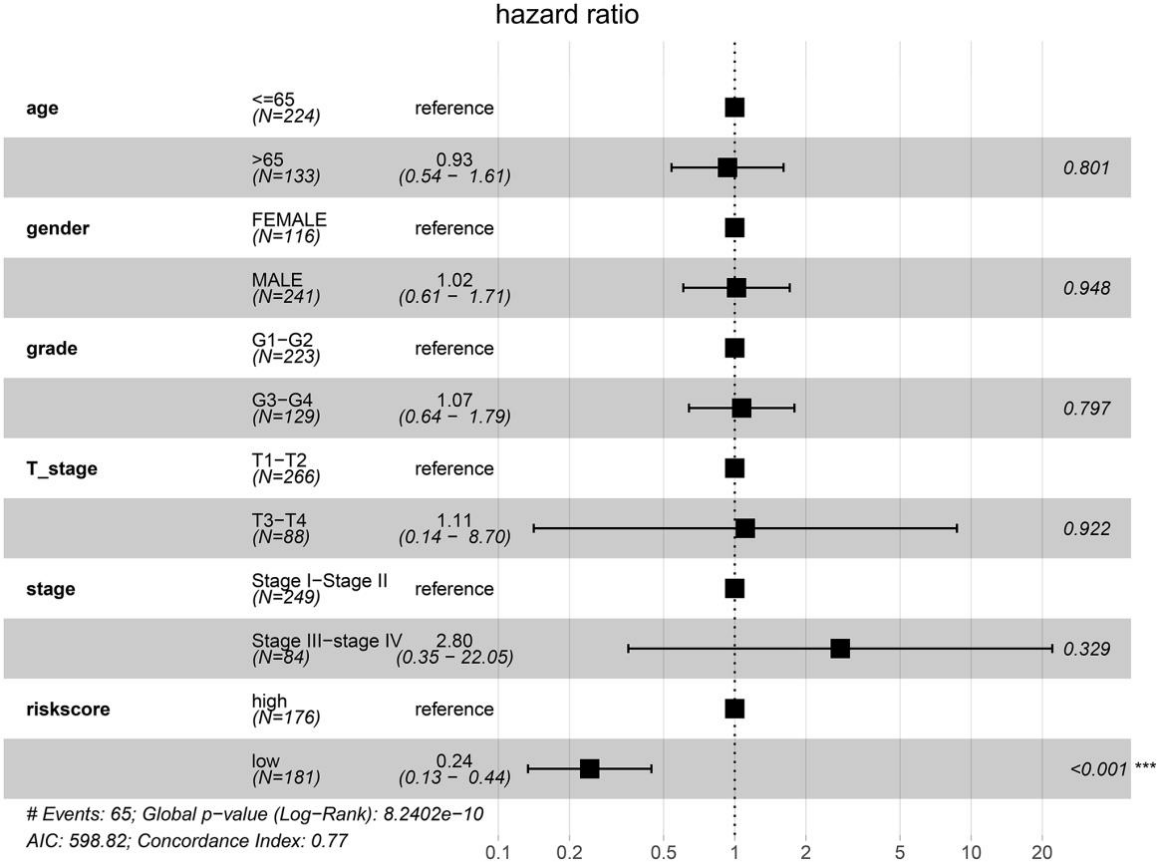
Low-risk of HCC patients from the TCGA cohort is an independent prognostic factor for disease-specific survival (DSS).

After controlling for the impact of clinical factors such as pathological stage and histological grade via multivariate Cox regression analysis, the prognosis of the low-risk group remained better than that of the high-risk group (hazard ratio [HR] = 0.24; 95% confidence interval [CI]: 0.13–0.44; *P* < 0.001) (Figure 10).

**Figure 11:**
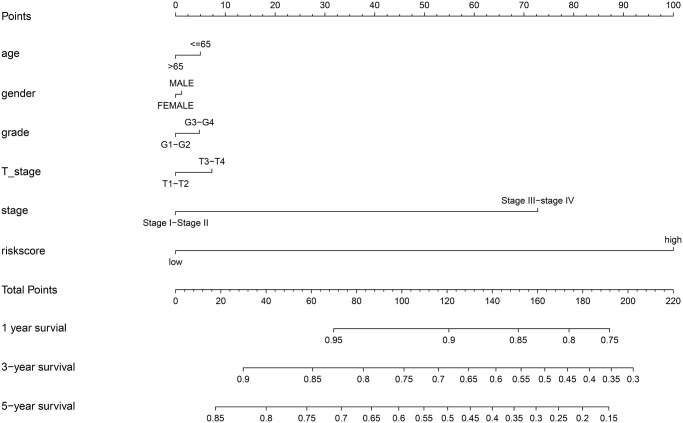
Nomogram to predict the probability of disease-specific survival (DSS) at 1, 3, and 5 years for HCC patients in the TCGA cohort.

We also constructed a nomogram that integrated risk score and clinical data including pathological stage and histological grade to predict the prognosis of HCC patients in the TCGA cohort more precisely, with a C-index = 0.768(95% CI: 0.712–0.824). Based on the score for each variable on the point scale of this nomogram, the probability of survival at 1, 3, and 5 years can be determined by calculating the total score (Figure 11).

**Figure 12:**
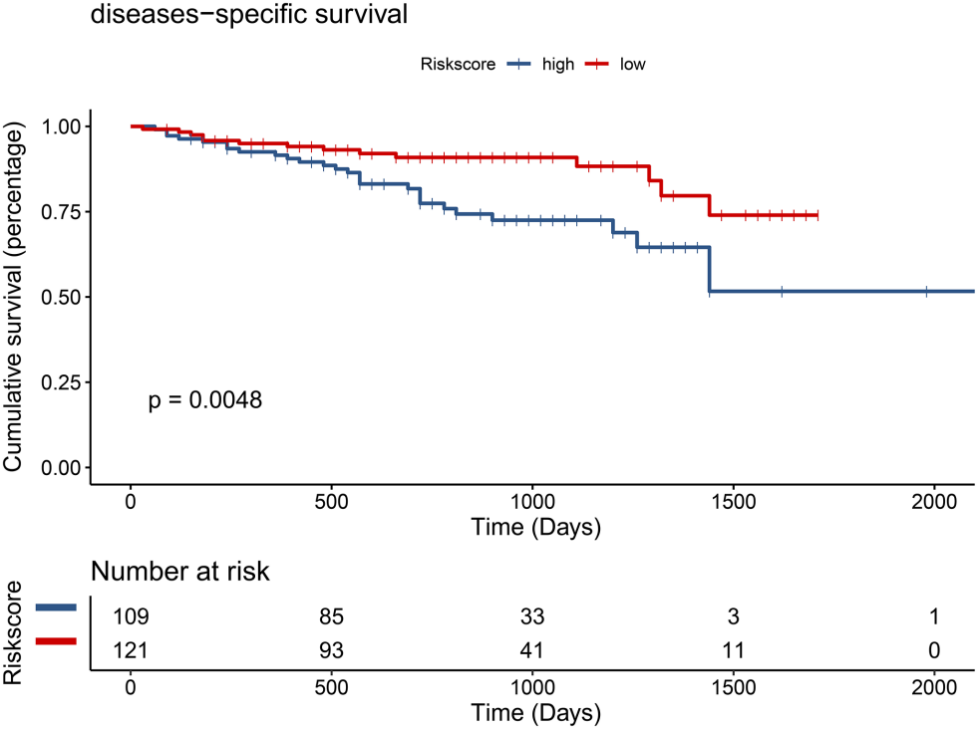
Validation of the prognostic model in the ICGC cohort by univariate Cox analysis.

**Figure 13:**
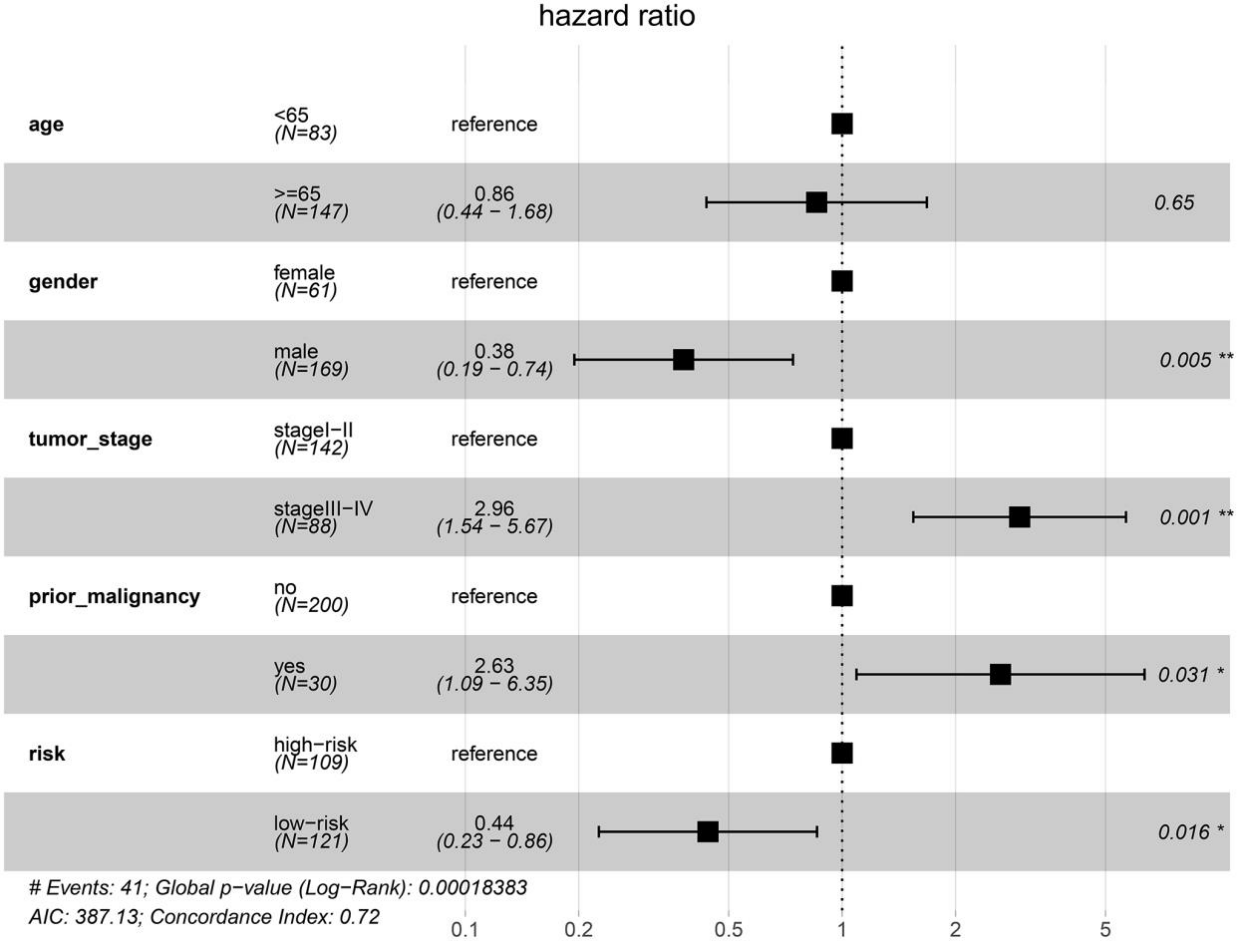
Validation of the prognostic model in the ICGC cohort by multivariate Cox analysis.

To examine whether the riskscore model was accurate in different cohorts, the same cut-off values of the 12 genes were used to calculate the risk scores of the 235 HCC patients in the ICGC cohort. The patients were divided into high-risk and low-risk groups. Consistent with the findings of the TCGA cohort, patients in the low-risk group had a better overall survival (OS) than patients in the high-risk group (*P* = 0.0048) (Figure 12). The difference remained significant after controlling for clinical factors (HR = 0.44, 95% CI: 0.23–0.86, *P* = 0.016) (Figure 13).

Taken together, the results indicate that the risk score can be used as an independent prognostic factor for patients with HCC.

## DISCUSSION

In recent years, more research has been devoted to examining the relations between energy metabolism and human malignancy. Numerous studies have focused on the impact of glycolysis on HCC progression, while the clinical significance of aerobic respiration has not been deeply explored. In this study, we compared gene expression from matched tumor and normal tissues in TCGA and ICGC cohorts, and identified 15 DEGs. After setting optimal cut-off points, univariate Cox regression analysis showed that all 15 genes were significantly associated with DSS in HCC patients from the TCGA cohort. Finally, based on the LASSO estimation [10], we chose 12 genes to construct a prognostic model.

Many studies have attempted to develop prognostic models for HCC patients. Chen et al. developed a 4-gene signature model to predict the prognosis of HCC patients, with a model formula that is more concise than ours [11]. However, the AUC or C-index was not provided in that study, and thus it is difficult to evaluate the quality of the model. A prognostic model for patients with HCC undergoing radiofrequency ablation (RFA) was recently reported [12]. The model incorporated tumor size and number, alpha fetoprotein (AFP) level (ng/ml), protein induced by vitamin K absence (PIVKA) level (mAU/ml), lymphocyte count (/ul), serum albumin level (g/dl), and ascites, and a nomogram was developed to predict patient survival. However, the C-index of the model (0.759) was a little lower than that of our model (0.768). As one of main metabolic features of tumor cells, genes involved in glycolysis have been screened to establish a six gene signature for HCC patients [13]. The diagnostic efficacy of the prognostic model was good (AUC = 0.765), but the prognostic value was not evaluated. We also tried to develop a prognostic model based on both glycolysis and aerobic respiration, but the result seemed to be unsatisfactory (Supplementary Figure 2), which might be mainly explained by the high heterogeneity of aerobic respiration and glycolysis pathways.

Most of the genes included in our prognostic model have been reported to be associated with survival in many different malignancies. *ACAA1* may be a marker for non-small cell lung cancer (NSCLC) diagnosis and prognosis, and may provide new insights for NSCLC treatment [14]. Up-regulation of *ACAT1* expression is involved in the progression of colorectal cancer (CRC) [15, 16]. Breast cancer growth and bone metastasis are prevented by silencing of *ATP6V1C1*, suggesting a potential therapy target [17]. *BDH2* is a tumor suppressor gene in HCC that regulates apoptosis and autophagy [18]. Up-regulation of *ECHS1* may promote cell proliferation in HCC [19], which is different than the results or our study. The difference of results may be explained by the sample divergence: *ETDFH* expression level has been found to be associated with AFP level, and it may also be an independent prognostic factor in HCC patients [20]. *GSTZ1* acts as a tumor suppressor gene in HCC, and *GSTZ1* deficiency might accelerate HCC progression [21]. *ME1* has been reported to be associated with poor survival in gastric cancer patients, and it may be an oncogene [22]. Down-regulation of *OGDHL* expression is correlated with poor prognosis in patients with pancreatic cancer [23]. *PCK1* suppressed HCC through promoting TCA cataplerosis, oxidative stress, and apoptosis in HCC cells [24]. Low expression of *PCK2* has been observed in osteosarcoma patients with metastasis and recurrence, and is associated with poorer survival [25]. *SLC16A3* has been shown to be a possible biomarker for prognosis of pancreatic cancer [26].

In this study, HCC patients identified in TCGA were dichotomized into a high-risk group and low-risk group by the risk score. Univariate and multivariate Cox regression analysis both indicated that the low-risk group had better DSS. We also validated the prognostic model in an ICGC cohort and obtained similar results. Based on the development of the prognostic model, low-risk was defined as a risk score < –0.6943, and high-risk was defined as a risk score > –0.6943. Overall, patients in the low-risk group had a better prognosis than patients in high-risk group, and a relatively precise survival probability could be calculated for each patient using the nomogram developed in the study. As such, the prognostic model may be used for the risk stratification in HCC patients, and potentially guide individualized patient treatments.

Several factors may have potentially influenced the outcome of this research. First, batch effects are important source of error in research based on RNA-sequencing data. In this study, we convert the format of RNA-sequencing data from fragments per kilobase of exon model per million mapped fragments (FPKM) to transcripts per kilobase of exon model per million mapped reads (TPM), which removed the impact of different sequencing depths. Second, and importantly, we identified DEGs from paired tumor and normal tissues, because we thought tumor and normal tissues from the same patient were comparable. Third, we used DSS as the prognostic indicator, which removed the impact of death from other factors to some extent. In the ICGC cohort, only 1 survival indicator was provided, so we empirically excluded patients with survival < 30 days to remove patients who died from other causes.

Some limitations of this study must be pointed out. We did not explore the possible mechanisms by which the genes identified serve a prognostic role. Besides, there is the potential of different sequencing data from different sequencing platforms; however, the prognostic model exhibited ideal performance based on Illumina platform. While the results of this study are promising, they need to be validated in other studies with larger numbers of patients and different sequencing platform. Finally, our study was a retrospective research, so it was difficult to prevent some inherent bias.

In conclusion, this study constructed and validated a prognostic model of HCC through a comprehensive analysis of genes involved in aerobic respiration. Based on risk score, HCC patients could be stratified into high-risk and low-risk groups, which may assist in developing individualized treatments for patients with HCC.

## METHODS

### Data acquisition and processing

RNA-sequencing and clinical data were downloaded from the LIHC project of The Cancer Genome Atlas (TCGA) data portal (https://portal.gdc.cancer.gov/), and the LIRI-JP project of International Cancer Genome Consortium (ICGC) data portal (https://dcc.icgc.org/releases/current/Projects/LIRI-JP).

A total of 285 genes were obtained for the citric acid cycle (TCA) and for respiratory electron transport (R-HSA-1428517) in Reactome (https://reactome.org/PathwayBrowser/#/R-HSA-1428517&PATH=R-HSA-1430728&DTAB=MT). Oxidative phosphorylation hallmark gene sets and KEGG_CITRATE_CYCLE_TCA_CYCLE of KEGG gene sets were obtained from the molecular signatures database (https://www.gsea-msigdb.org/gsea/msigdb/collections.jsp#C5).

For calculations, the format of RNA-sequencing data was converted from fragments per kilobase of exon model per million mapped fragments (FPKM) to transcripts per kilobase of exon model per million mapped reads (TPM).

### Differential and functional analysis

The “edgR” package in R software was used to analyze the raw RNA-sequencing data counts from TCGA and the ICGC with the criteria of: |log_2_ fold-change| > 1.0, and false discovery rate (FDR) adjusted to *P* < 0.05. A total of 15 overlapping differentially expressed genes (DEGs) associated with aerobic respiration were identified. Volcano plots and heat maps of the DEGs were generated to visualize the results using the “ggpubr” package and the “pheatmap” package. To understand the biological processes of the DEGs, functional enrichment analysis of the DEGs was performed with the “clusterProfile” package [27]. Gene Ontology (GO) and Kyoto Gene and Genomic Encyclopedia (KEGG) analyses were conducted to assess relevant functional categories based on the threshold of *P* < 0.05.

### Construction of the prognostic model and model validation

To eliminate patients who might die of non-cancer-related diseases, we included 357 patients from TCGA who were followed-up for more than 1 day with clear disease-specific survival (DSS) data, and 235 patients from the ICGC who were followed-up for more than 30 days with clear survival status data. The optimum cut-off point of DEG expression was determined using the “surv_cutpoint” function of the “survminer” package in R. Ideal DEGs with a significance level of *P* < 0.05 were selected through univariate Cox regression analysis using the “survival” package in R. The LASSO function of the “glmnet” package in R was used to select the most useful prognostic genes among the DEGs. The most useful prognosis-related DEGs were selected based on LASSO estimation, and the optimal value for penalization coefficient lambda was chosen after running cross-validation likelihood (cvl) 1,000 times [10]. Subsequently, the Cox coefficients and expression values of the DEGs were extracted to calculate the risk score. The expression value of a gene, which was considered highly expressed, was defined as 0, and the expression value of a gene which was considered to have low expression was defined as 1.

A formula for the prognostic model was established to predict patient survival based on the formula:

Risk score = ∑Cox coefficient of gene Xi × expression value of gene Xi. The sensitivity and specificity of survival prediction based on the risk score was calculated, and receiver operating characteristic (ROC) curve analysis was performed using the “survivalROC” package in R. We also used multivariate Cox regression analysis to control for the impact of potential confounding factors, and Forest plots and a nomogram were constructed using the “rms” package and “ggforest” functions in the “survminer” package of R. The C-index of the prognostic model was calculated using the “rcorrcens” function. The prognostic model was validated using the ICGC cohort and the same cut-off points.

Overall, we developed a flow chart to help readers understand the steps of our manuscript (Supplementary Figure 3).

## Supplementary Materials

Supplementary Figures
