# Supplementary material for: Development of a prognostic model for hepatocellular carcinoma using genes involved in aerobic respiration
Source: Aging (Albany NY). 2021 Apr 26;13(9):13318–32. doi: 10.18632/aging.203021 (PMC8148449; doi:10.18632/aging.203021)
Supplement: Supplementary Figures [file aging-13-203021-s001.pdf]

SUPPLEMENTARY FIGURES

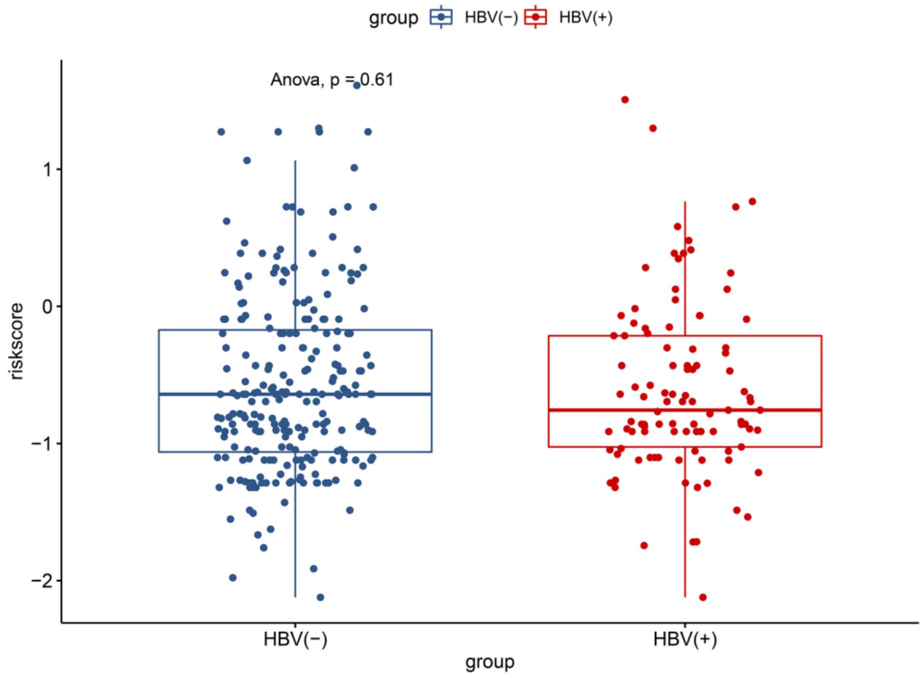

Supplementary Figure 1. No significant differences of risk score were found between HBV-related HCC and non-HBV HCC.

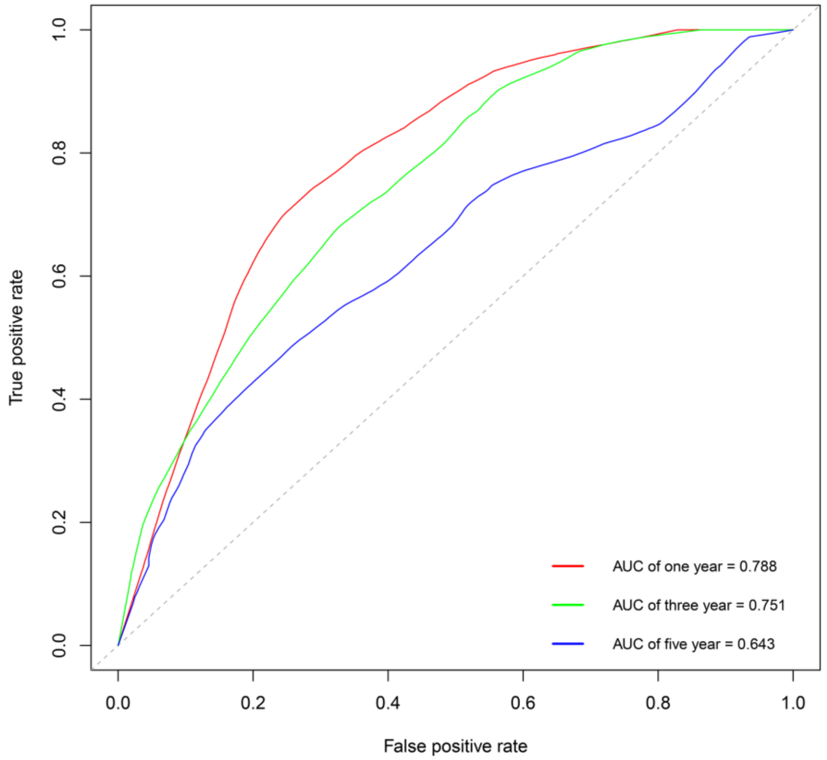

Supplementary Figure 2. Survival-dependent receiver operating characteristic (ROC) curves showed the prognostic value of the prognostic model based on genes of aerobic respiration and glycolysis.

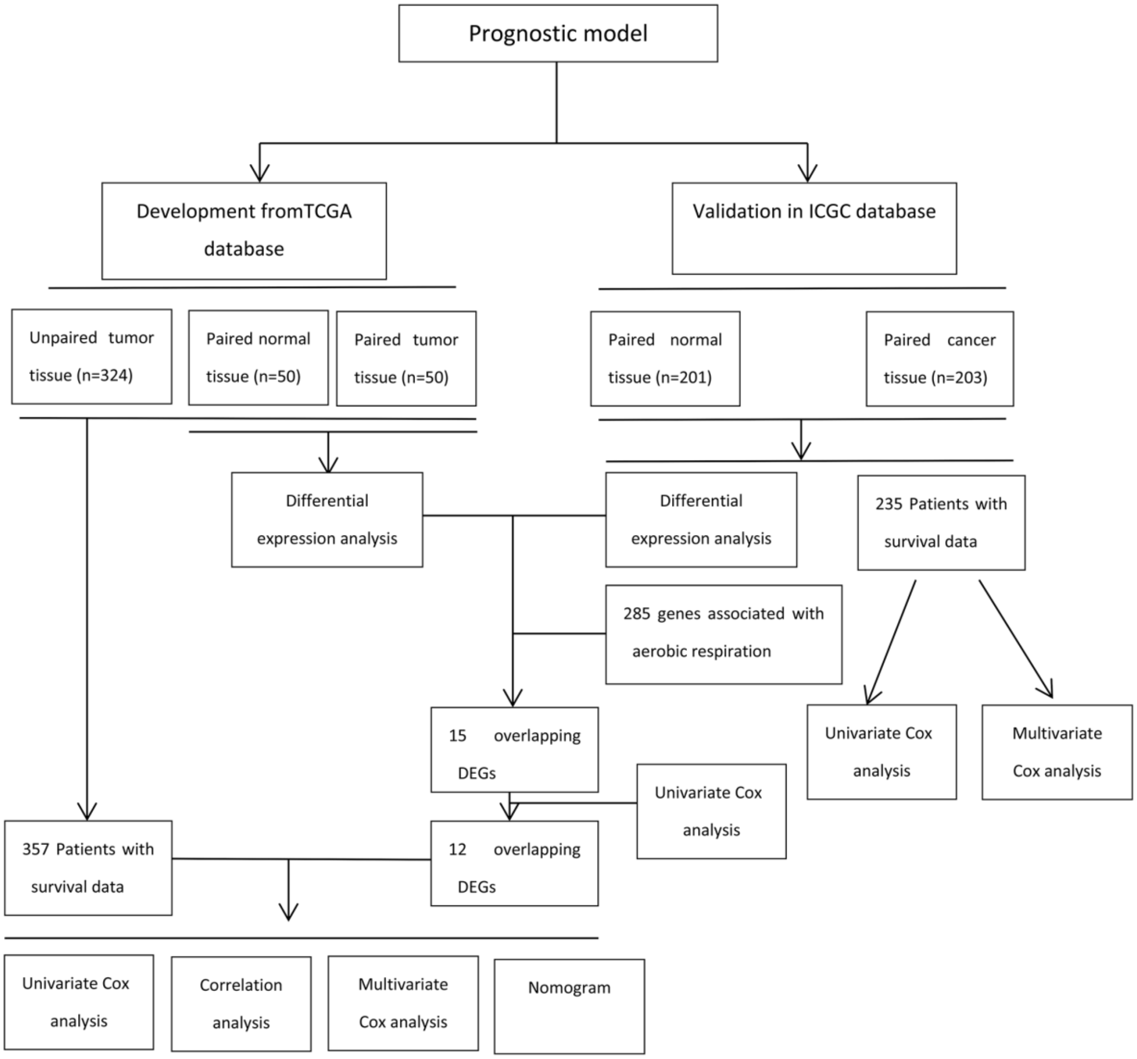

Supplementary Figure 3. Flow chart of establishing a prognostic model based on 12 genes for HCC.
